# Prediction of the debulking effect of rotational atherectomy using optical frequency domain imaging: a prospective study

**DOI:** 10.1007/s12928-023-00928-9

**Published:** 2023-04-05

**Authors:** Tomoyo Hamana, Hiroyuki Kawamori, Takayoshi Toba, Makoto Nishimori, Kosuke Tanimura, Shunsuke Kakizaki, Koichi Nakamura, Daichi Fujimoto, Satoru Sasaki, Yuto Osumi, Masayoshi Fujii, Seigo Iwane, Tetsuya Yamamoto, Shota Naniwa, Yuki Sakamoto, Yuta Fukuishi, Koshi Matsuhama, Ken-ichi Hirata, Hiromasa Otake

**Affiliations:** 1 Division of Cardiovascular Medicine, Department of Internal Medicine, Kobe University Graduate School of Medicine, 7-5-2 Kusunoki-Cho, Chuo-Ku, Kobe, Hyogo 650-0017 Japan; 2 Division of Epidemiology, Kobe University Graduate School of Medicine, Kobe, Japan

**Keywords:** Optical frequency domain imaging, Rotational atherectomy, Percutaneous coronary intervention, Prediction, Prospective study

## Abstract

This study determined the predictive accuracy of optical frequency domain imaging (OFDI) on debulking effects of rotational atherectomy (RA) and compared the predictive accuracy of OFDI catheter-based with Rota wire-based prediction methods. This prospective, single-center, observational study included 55 consecutive patients who underwent OFDI-guided RA. On pre-RA OFDI images, a circle, identical to the Rota burr was drawn at the center of the OFDI catheter (OFDI catheter-based prediction method) or wire (wire-based prediction method). The area overlapping the vessel wall was defined as the predicted ablation area (P-area). The actual ablated area (A-area) was measured by superimposing the OFDI images before and after RA. The overlapping P-area and A-area were defined as overlapped ablation area (O-area), and the predictive accuracy was evaluated by %Correct area (O-area/P-area) and %Error area (A-area  − O-area/A-area). The median %Correct and %Error areas were 47.8% and 41.6%, respectively. Irrelevant ablation (low %Correct-/high % Error areas) and over ablation (high %Correct-/high % Error areas) were related to deep vessel injury and intimal flap outside the P-area. The predictive accuracy was better in the OFDI catheter-based prediction method than the wire-based prediction method in the cross sections where the OFDI catheter and wire came in contact. However, it was better in the latter than the former where the OFDI catheter and wire were not in contact. OFDI-based simulation of the RA effect is feasible though accuracy may be affected by the OFDI catheter and wire position. OFDI-based simulation of RA effect might reduce peri-procedural complications during RA.

**Supplementary Information:**

The online version contains supplementary material available at 10.1007/s12928-023-00928-9.

## Background

Rotational atherectomy (RA) is an established treatment option in percutaneous coronary intervention (PCI) for severely calcified lesions. It contributes to the successful delivery of treatment devices and optimal stent expansion [1]. However, RA has a potential risk of procedural complications, including perforation, coronary dissection, and slow-flow phenomenon, leading to fatal situations [2].

Optical frequency domain imaging (OFDI) is a high-resolution imaging device that enables precise assessment of intracoronary findings [3, 4]. The primary role of preoperative OFDI imaging in RA cases is to evaluate the indication, effectiveness, and safety of RA in severely calcified cases [5]. Previous reports demonstrated that OFDI could accurately predict the debulking effects of RA under several specific conditions such as floppy wire use, narrow lumen area compared with RA burr size, and the closer placement of OFDI catheter to the intima [6]. However, the overall predictive accuracy was not very high. Furthermore, the previous study had several limitations owing to its retrospective nature. Additionally, the previous study demonstrated that the predictive accuracy of the OFDI catheter-based prediction method decreased when the OFDI catheter is distant from the wire, suggesting that wire-based (not OFDI catheter based) predictions might be favorable in such conditions. Thus, this novel study aimed to determine the predictive accuracy of OFDI on debulking effects of RA with a prospective protocol and compared the predictive accuracy between OFDI catheter-based prediction and guidewire-based prediction methods.

## Methods

### Study design and population

In this single-center, observational study, we prospectively enrolled consecutive patients who fulfilled the following criteria. The eligibility criteria were: (1) patients ≥ 20 years old; (2) patients with severe calcified lesions; (3) patients who were scheduled to undergo OFDI-guided RA from August 2019 to August 2022 at our institution. Severe calcified lesions are defined as radiopacity observed without cardiac motion, visible on both sides of the arterial lumen, as a double track in accordance with previous study [7]. The exclusion criteria were patients with: (1) acute coronary syndrome; (2) cardiac shock; (3) in-stent restenosis; (4) chronic total occlusion; (5) coronary artery bypass grafted lesions; (6) poor image quality; and (7) those who could not have OFDI passed before RA. All participants provided written informed consent before PCI procedures. They were subsequently treated according to the following protocols and their peri-procedural information was prospectively collected. This study protocol complied with the ethical standards as laid down in the 1964 Declaration of Helsinki and its later amendments. The study was approved by the Ethics Committee of Kobe University Hospital and was registered in the University Hospital Medical Information Network Clinical Trial Registry (UMIN 000035634).

### Procedures

PCI procedure was performed using 6Fr to 8Fr catheters through the femoral or radial artery. After an intracoronary bolus injection of nitroglycerin, OFDI was performed before RA using a 0.014-inch conventional guidewire; if the OFDI catheter could not pass through the target lesion, pre-dilatation with a 2.0-mm or smaller balloon was permitted. After replacing the conventional wire with a 0.009-inch RotaWire (Boston Scientific Corporation, Natick, MA, USA) using a microcatheter, OFDI was re-performed. The choice of RotaWire floppy or extra-support was left to the discretion of the operator. RA was performed using the Rotalink Plus rotational atherectomy system (Boston Scientific Corporation) at a rotation speed of 170,000 to 200,000 rpm according to the standard practice [1, 2]. During the RA procedure, a drug cocktail containing heparin, atropine, nitroglycerin, and nicorandil was administered to prevent slow flow and no-reflow phenomena. The first chosen burr size was based on achieving a burr to artery ratio < 0.7. Detailed procedural information about starting speed, max deceleration speed, total debulking counts, and total run time was prospectively recorded.

### Complications

The slow flow was defined as reduced coronary flow with Thrombolysis In Myocardial Infarction (TIMI) grades ≤ 2 instantaneously after RA. No reflow was defined as cessation of blood flow to the distal coronary artery immediately after RA [8]. Perforation was defined as type III perforation due to the RA burr [9].

### OFDI imaging and analysis

OFDI system (LUNAWAVE, Terumo, Tokyo, Japan) and OFDI catheter (FastView, Terumo) was used in all cases. After manual calibration, the OFDI catheter was advanced as distally as possible to the ostium of each vessel. It was strongly recommended to carefully remove deflection before OFDI imaging to prevent the separation of the OFDI catheter and wire as much as possible. For image acquisition, blood in the lumen was replaced with contrast media or low molecular dextran. OFDI imaging core was pulled back at a rate of 40 mm/s using a standalone electric-controlled pullback monitor.

Offline OFDI analysis was performed using a dedicated workstation (LUNAWAVE Offline Viewer, Terumo, Tokyo, Japan). The cross-sectional OFDI images were matched before and after RA based on the location of the intravascular structure and side branches. For pre-RA images, cross-sectional images with Rota wire were selected. Target cross sections for OFDI analysis were determined from the beginning to the end of the cross-section where the debulking effect of RA was visually confirmed. Additionally, the lumen diameter and lumen area were measured before and after RA. To evaluate the severity of calcification, the arc and minimum depth of calcium were measured. The calcification types were classified into three groups: circumferential (an angle of calcification of 270° or more); eccentric (an angle of calcification of 270° or less); and nodular (the protrusion of calcification into the lumen). To assess the position of Rota wire and OFDI catheter, the minimum distances between: OFDI catheter and intima; wire and intima; wire and OFDI catheter, were measured before RA.

### OFDI catheter-based prediction method

**Fig. 1:**
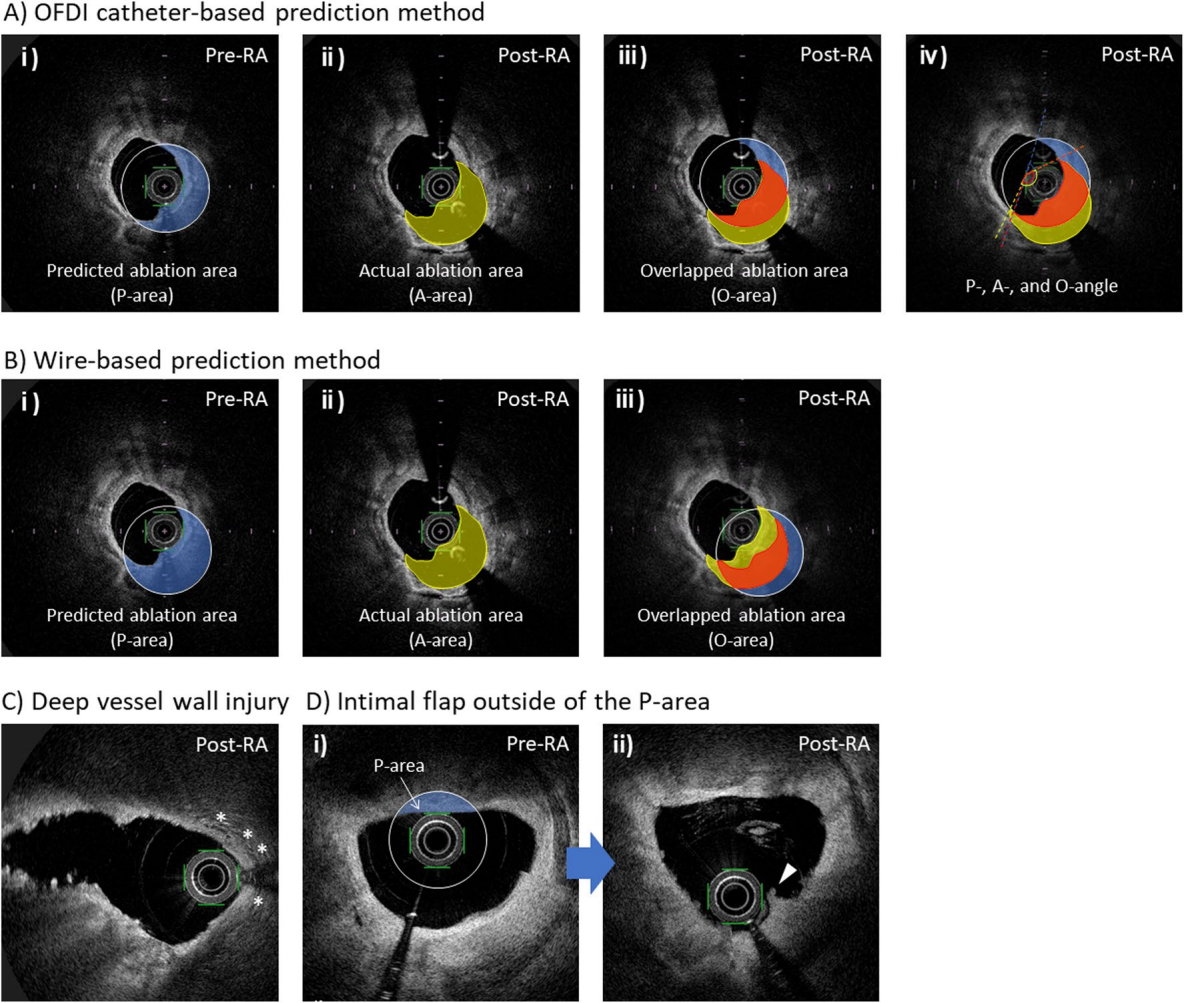
Definitions of predicted, actual, and overlapped ablation area/angle, and OFDI findings before and after RA. **A** OFDI catheter-based prediction method. **B** Wire-based prediction method. Cross-sectional OFDI image before and after RA. A white circle identical to the Rota burr size (2.0-mm) was drawn at the center of the OFDI catheter or the wire (**A**, **B**-i). The blue area indicates the predicted ablation area (P-area) (**A**, **B**-ii). The yellow area denotes the actual ablation area (A-area) (**A**, **B**-iii). The red area, where the P-area and A-area overlapped is defined as overlapped ablation area (O-area) (**A**, **B**-iv). Blue, yellow, and red angles signify the P-, A-, and O-angles, respectively (**A**-iv). **C** Deep vessel wall injury. Asterisk shows deep vessel injury extending beyond the media following RA. **D** Intimal flap outside of the P-area. D-1 and D-2 are corresponding cross sections before and after RA. The white arrowhead suggests an intimal flap outside the P-area (the white circle indicates the Burr size circle). *OFDI* optical frequency domain imaging; *RA* rotational atherectomy

The OFDI analysis was performed at 1-mm intervals by dedicated independent observers (T.H. and M.F.) blinded to the target lesion features, as in our previous retrospective study [6]. On the pre-RA OFDI images, a circle of identical size to the Rota burr was drawn at the center of the OFDI catheter. The area overlapping the vessel wall was defined as the predicted ablation area (P-area, Fig. 1A-i). The actual ablated area was measured by superimposing the OFDI images before and after RA (A-area, Fig. 1A-ii). The overlap of the P-area and A-area was defined as overlapped ablation area (O-area, Fig. 1A-iii). In addition, the angles of the P-, A-, and O-areas (P-, A-, and O-angles, respectively) around the lumen center were measured on the corresponding OFDI images (Fig. 1A-iv). The P-, A-, and O-volumes were calculated by integrating the P-, A-, and O-areas, respectively. %Correct area was computed as O-area divided by P-area, and %Error area was calculated as A-area minus O-area divided by A-area. Similarly, %Correct and %Error angles and %Correct and %Error volumes were calculated. In each cross-section after RA, the presence of deep vessel injury extending beyond the media (Fig. 1C) and intimal flap outside the P-area (Fig. 1D) were evaluated.

### Wire-based prediction method

In this prospective study, another prediction method involved drawing a circle at the center of the wire instead of drawing a circle at the center of the OFDI catheter, with subsequent analysis in the same manner (Fig. 1Bi–iii). This new analysis method is referred to as the ‘wire-based prediction method’ and the previous method as the ‘OFDI catheter-based prediction method.’ All areas and angles were measured using Image J software (version 1.53 k; US National Institutes of Health, Bethesda, MD, USA).

### Statistical analyses

Categorical variables are presented as numbers (percentages) and compared with a chi-square test or Fisher’s exact test. Continuous variables were expressed as mean ± standard deviation (SD) or median (interquartile range [IQR]) and compared using the Student’s *t*-test or the Mann–Whitney U test based on their distributions. Logistic regression analysis was used to identify independent factors associated with prediction ability. Variables of OFDI data with *P* < 0.15 in the univariable analysis were included in the multivariable analysis. The results were presented as odds ratio (OR) with a 95% confidence interval (CI). Receiver operating characteristic (ROC) analysis was used to determine the optimal cut-off value of the minimum distance between the OFDI catheter and wire associated with irrelevant ablation. All statistical analyses were performed using MedCalc software program version 19.8 (MedCalc Software Ltd, Ostend, Belgium); a two-sided *P* < 0.05 was considered statistically significant.

## Results

### Study population and baseline characteristics

From 60 patients (60 lesions) who fulfilled the eligibility criteria, 4 patients who could not have OFDI passed before RA and 1 patient with poor image quality were excluded. Finally, a total of 55 patients (55 lesions) who successfully underwent OFDI imaging before and immediately after RA were analyzed (Supplemental Fig. 1). Patient, lesion, procedural, and angiographic characteristics and complications were described in Table 1.

**Table 1:** Patient, lesion, and procedural characteristics

	Overall
Patients, *n*	55
Age, years	74.0 ± 8.7
Male, *n* (%)	48 (87.3)
Comorbidities	
Hypertension, *n* (%)	42 (76.4)
Diabetes mellitus, *n* (%)	38 (69.1)
Dyslipidemia, *n* (%)	39 (70.9)
Chronic kidney disease, *n* (%)	42 (76.4)
Hemodialysis, *n* (%)	16 (29.1)
Lesion, *n*	55
Cross-section, *n*	474
Target vessel	
LAD, *n* (%)	33 (60.0)
LCx, *n* (%)	12 (21.8)
RCA, *n* (%)	10 (18.2)
Target lesion	
Proximal, *n* (%)	33 (60.0)
Mid, *n* (%)	20 (36.4)
Distal, *n* (%)	2 (3.6)
Approach site	
Transfemoral, *n* (%)	34 (61.8)
Transradial, *n* (%)	21 (38.2)
Guiding catheter type	
Judkins, *n* (%)	11 (20.0)
Extra back up, *n* (%)	31 (56.4)
Amplatzer, *n* (%)	13 (23.6)
Rotawire type	
Floppy wire, *n* (%)	38 (69.1)
Extra-support wire, *n* (%)	17 (30.9)
First burr size	
1.50 mm, *n* (%)	19 (34.5)
1.75 mm, *n* (%)	26 (47.3)
2.00 mm, *n* (%)	8 (14.5)
2.15 mm, *n* (%)	1 (1.8)
2.25 mm, *n* (%)	1 (1.8)
Start speed, × 10^4^	18.7 ± 0.80
Max deceleration speed, rpm	4000 (3000–6000)
Total debulking counts	22 (16–27)
Total run time, sc	93 (56–116)
Complications	
Slow flow, *n* (%)	10 (18.2)
No reflow, *n* (%)	4 (7.3)
Perforation, *n* (%)	0 (0.0)
Burr stuck, *n* (%)	0 (0.0)

Values are expressed as average ± standard deviation, median (interquartile range) or *n* (%)

*LAD* left anterior descending; *LCx* left circumflex; *RCA* right coronary artery

### OFDI measurement before and after RA

Table 2 shows OFDI measurement before and after RA performed using the “OFDI catheter-based prediction method.” A total of 474 cross sections were analyzed. In the cross-section-based analysis, the median %Correct and %Error angles were 69.8% and 15.6%, respectively, and the median %Correct area and %Error areas were 47.8% and 41.6%, respectively. Pre- and post-operative lumen measurement, severity and morphology of calcification, and OFDI catheter and wire positions were also depicted in Table 2. Deep vessel injury extending beyond media, and intimal flap outside of the P-area was observed in 3.8% and 10.5% of the total debulked cross sections. On a patient-basis, the median %Correct and %Error volumes were 44.1% and 42.1%, respectively (Table 2).

**Table 2:** OFDI measurements before and after RA

Cross-section-based analysis	*n* = 474
Pre/post-operative lumen measurements	
Pre minimum lumen diameter, mm	1.56 (1.24–2.03)
Pre mean lumen diameter, mm	1.93 (1.60–2.43)
Pre lumen area, mm²	2.91 (2.00–4.60)
Burr/pre lumen ratio	0.90 (0.73–1.12)
Post minimum lumen diameter, mm	1.74 (1.53–2.10)
Post mean lumen diameter, mm	2.18 (1.88–2.60)
Post lumen area, mm²	3.70 (2.80–5.30)
Degree and morphology of calcification	
Arc of calcium, °	238 (180–307)
Minimum depth of calcium, µm	430 (300–640)
Circumferential calcification, *n* (%)	172 (36.3)
Eccentric calcification, *n* (%)	243 (51.3)
Nodular calcification, *n* (%)	59 (12.4)
RA-related measurement	
Angle-based analysis	
P-angle, °	143 (87–232)
A-angle, °	123 (85–180)
O-angle, °	89 (57–156)
%Correct angle, %	69.8 (48.9–87.5)
%Error angle, %	15.6 (3.7–34.0)
Area-based analysis	
P-area, mm²	0.74 (0.47–1.02)
A-area, mm²	0.67 (0.36–1.09)
O-area, mm²	0.34 (0.16–0.55)
%Correct area, %	47.8 (28.6–66.9)
%Error area, %	41.6 (18.0–66.8)
OFDI catheter and rota-wire position	
Minimum distance between the OFDI catheter and intima, mm	0.00 (0.00–0.09)
Minimum distance between the wire and intima, mm	0.21 (0.11–0.38)
Minimum distance between the OFDI catheter and wire, mm	0.00 (0.00–0.00)
OFDI findings following RA	
Deep vessel wall injury extending beyond media, *n* (%)	18 (3.8)
Intimal flap outside of the P-area, *n* (%)	50 (10.5)

Patient-based analysis	*n* = 55
Pre/post-RA lumen measurements	
Pre minimum lumen area, mm^2^	1.80 (1.20–2.30)
Pre mean lumen area, mm²	3.15 (2.11–3.96)
Post minimum lumen area, mm^2^	2.58 (2.12–3.10)
Post mean lumen area, mm^2^	3.94 (3.03–4.79)
RA-related measurement	
Angle-based analysis	
Mean P-angle, °	158 (113–228)
Mean A-angle, °	134 (103–170)
Mean O-angle, °	101 (66–149)
Mean %Correct angle, %	64.6 (48.4–73.6)
Mean %Error angle, %	18.8 (10.7–39.1)
Volume-based analysis	
P-volume, mm^3^	5.91 (2.36–10.9)
A-volume, mm^3^	4.84 (2.63–9.48)
O-volume, mm^3^	2.42 (0.89–5.03)
%Correct-volume, %	44.1 (37.2–57.3)
%Error-volume, %	42.1 (23.6–61.2)

Values are expressed as median (interquartile range) or n (%). Burr/lumen ratio was calculated by dividing the Rota burr size by the mean lumen diameter

*RA* rotational atherectomy; *OFDI* optical frequency domain imaging; *P-angle* angle of predicted ablation area; *A-angle* angle of actual ablation area; *O-angle* angle of overlap ablation area; *P-area* predicted ablation area; *A-area* actual ablation area; *O-area* overlap ablation area; *P-volume* predicted ablation volume; *A-volume* actual ablation volume; *O-volume* overlap ablation volume; *%Correct angle (area, volume)* percentage of correct ablation angle (area, volume); *%Error angle (area, volume)* percentage of error ablation angle (area, volume)

### Factors associated with a predictive accuracy of OFDI for RA effect

**Fig. 2:**
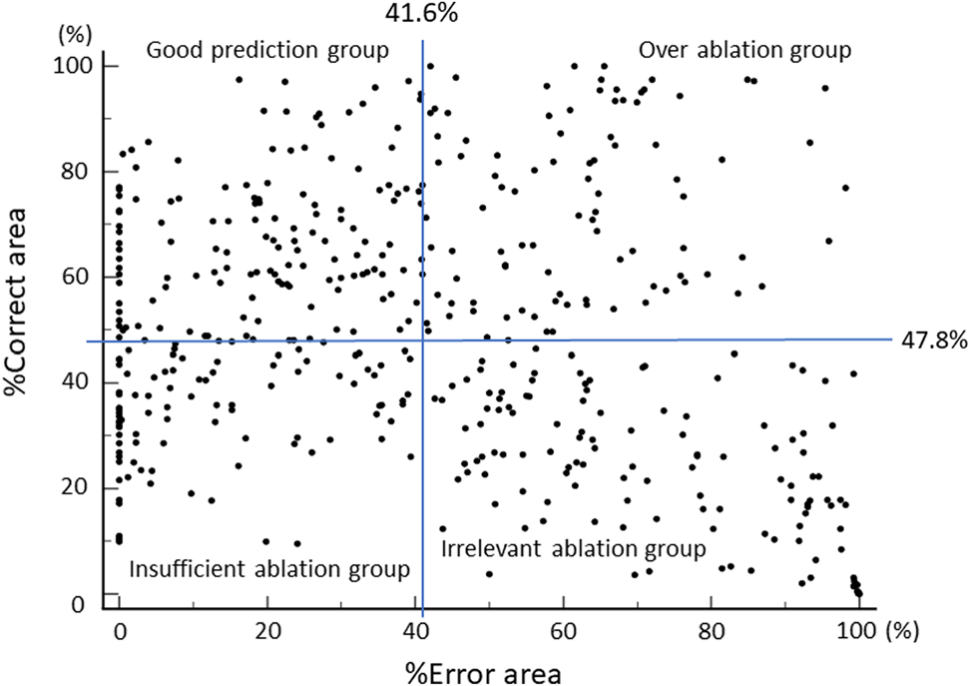
Distribution of %Correct- and %Error area in all cross sections. All cross sections are plotted according to %Correct- and % Error areas. The median %Correct- and %Error areas are 47.8%, and 41.6%, respectively. Cross-sections are divided into four groups: good prediction group (high %Correct/low %Error area), over ablation group (high %Correct/high %Error area), insufficient ablation group (low %Correct/low %Error area), and irrelevant ablation group (low %Correct/high %Error area)

All cross sections were divided into four groups based on the median %Correct area and % Error areas: good prediction group (high %Correct-/low %Error area); over ablation group (high %Correct-/high %Error area); insufficient ablation group (low %Correct-/low %Error area); and irrelevant ablation group (low %Correct-/high %Error area), as shown in Fig. 2. Representative cases of the four groups are described in Supplementary Fig. 2. The multivariable logistic regression analysis revealed that the short distance between the wire and intima (odds ratio [OR] 0.89, 95% CI 0.80–1.00, *P* = 0.042) and the short distance between the OFDI catheter and wire (OR 0.55, 95% CI 0.36–0.86, *P* = 0.008) was independently associated with the good prediction (Table 3). In contrast, the long distance between the: OFDI catheter and intima (OR 1.43, 95% CI 1.17–1.76, *P* = 0.001); wire and intima (OR 1.13, 95% CI 1.02–1.25, *P* = 0.020); OFDI catheter and wire (OR 1.72, 95% CI 1.32–2.25, *P* < 0.001) was independently associated with the irrelevant prediction (Table 4). Supplemental Tables 1 and 2 indicate that logistic regression analyses are associated with the good prediction group and irrelevant ablation group classified by the median %Correct and %Error volumes. In this lesion-based analysis, the multivariable logistic regression analysis revealed that the presence of left anterior descending lesions and right coronary artery lesions were independently associated with good prediction and irrelevant ablation groups, respectively.

**Table 3:** Univariable and multivariable logistic regression analyses according to the good prediction group

Variables	Univariable			Multivariable		
	OR	95% CI	*P* value	OR	95% CI	*P* value
Pre lumen area	0.93	0.84–1.01	0.095	–	–	–
Burr / lumen ratio	2.34	1.15–4.76	0.019	–	–	–
Distance between the OFDI catheter and intima (per 0.1 increase)	0.73	0.58–0.91	0.005	–	–	–
Distance between the wire and intima (per 0.1 increase)	0.86	0.78–0.96	0.005	0.89	0.80–1.00	0.042
Distance between the OFDI catheter and wire (per 0.1 increase)	0.53	0.34–0.82	0.004	0.55	0.36–0.86	0.008
Arc of calcium	1.00	1.00–1.00	0.58			
Thickness of calcium	0.80	0.29–2.26	0.68			

*OR* odds ratio; *CI* confidence interval; *OFDI* optical frequency domain imaging

**Table 4:** Univariable and multivariable logistic regression analyses according to the irrelevant ablation group

Variables	Univariable			Multivariable		
	OR	95% CI	*P* value	OR	95% CI	*P* value
Pre lumen area	1.03	0.95–1.12	0.48			
Burr / lumen ratio	0.72	0.35–1.49	0.38			
Distance between the OFDI catheter and intima (per 0.1 increase)	1.61	1.35–1.92	< 0.001	1.43	1.17–1.76	0.001
Distance between the wire and intima (per 0.1 increase)	1.18	1.08–1.30	< 0.001	1.13	1.02–1.25	0.020
Distance between the OFDI catheter and wire (per 0.1 increase)	1.77	1.38–2.27	< 0.001	1.72	1.32–2.25	< 0.001
Arc of calcium	1.00	1.00–1.00	0.90			
Thickness of calcium	0.57	0.21–1.56	0.27			

*OR* odds ratio; *CI* confidence interval; *OFDI* optical frequency domain imaging

### Impact of predictive accuracy of OFDI on post-RA findings

Supplemental Fig. 2 shows the prevalence of post-RA findings among the four groups. The incidence of deep vessel wall injury extending beyond the media was significantly higher in the over ablation group and the irrelevant ablation group than in the other groups (*P* for χ^2^ test = 0.008) (Supplemental Fig. 3A). Furthermore, the prevalence of intimal flap outside the P-area was significantly higher in the over ablation group and the irrelevant ablation group compared with other groups (*P* for χ^2^ test < 0.001) (Supplemental Fig. 3B).

**Fig. 3:**
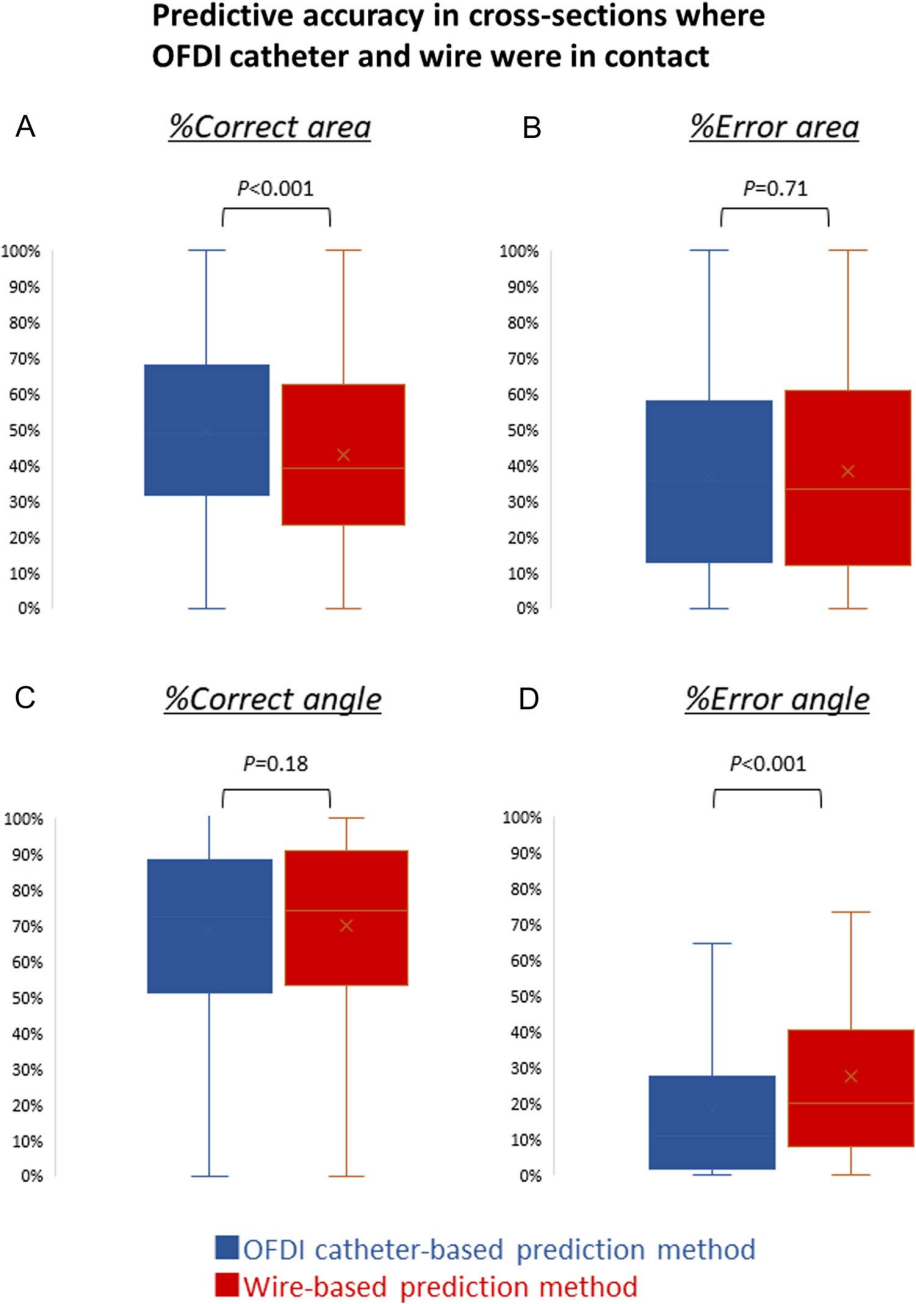
Predictive accuracy in cross sections where the OFDI catheter and wire are in contact. Comparison of **A** %Correct- and **B** %Error area and **C** %Correct- and **D** %Error angle between OFDI catheter-based and wire-based prediction method in cross sections where the OFDI catheter and wire are in contact. *OFDI* optical frequency domain imaging

### Comparison of OFDI catheter-based and wire-based prediction method

In the multivariable logistic regression analysis, a short distance between the OFDI catheter and wire was independently associated with a good prediction (Table 3). ROC curve analysis demonstrated the minimum distance between the OFDI catheter and wire that predicted cross-section with irrelevant ablation group had the cut-off value of > 0.0-mm (sensitivity: 40.0%, specificity: 83.0%, area under the curve: 0.620, *P* < 0.001) (Supplemental Fig. 4). Based on the results, all cross sections were divided into two groups based on whether or not the OFDI catheter and wire were in contact. Of the 474 cross sections, 357 cross sections (75.3%) showed the OFDI catheter and wire in contact. Subsequently, we compared OFDI catheter-based and wire-based prediction methods in each cross-sectional group.

**Fig. 4:**
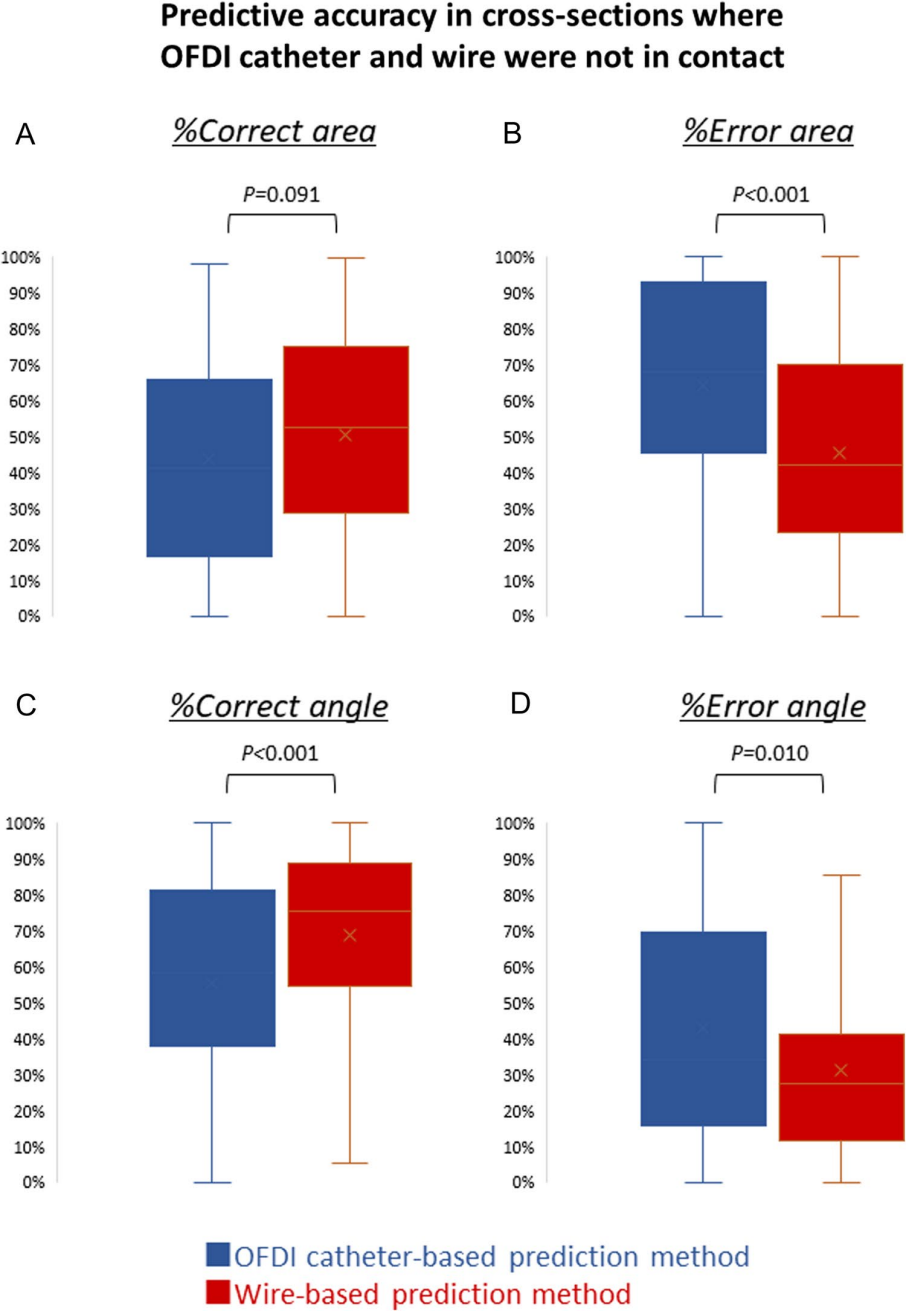
Predictive accuracy in cross sections where the OFDI catheter and wire are not in contact. Comparison of **A** %Correct- and **B** %Error area and **C** %Correct- and **D** %Error angle between OFDI catheter-based and wire-based prediction method in cross sections where the OFDI catheter and wire are not in contact. *OFDI* optical frequency domain imaging

**Fig. 5:**
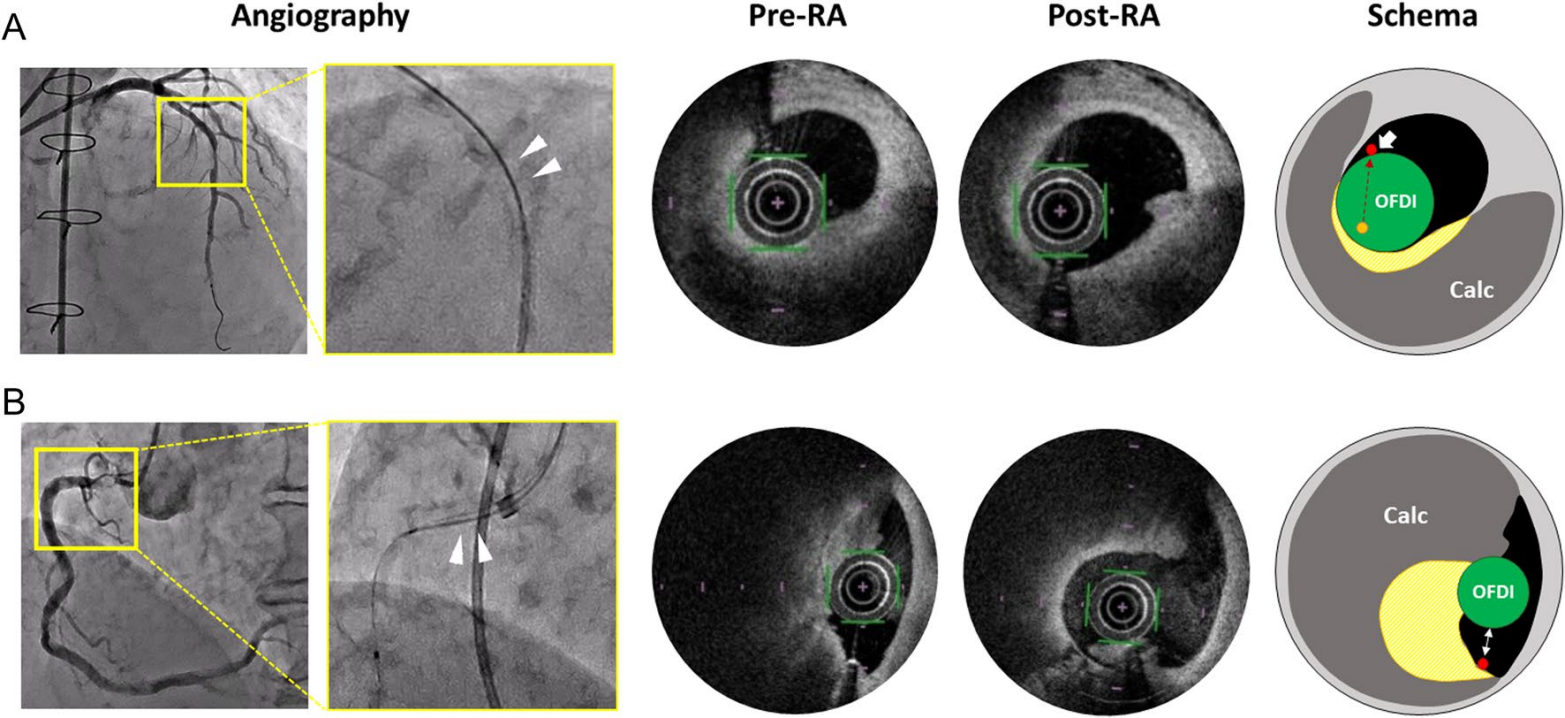
Representative cases. Angiography images with and without contrast (left). White triangles indicate the OFDI catheter and wire position of the target lesion. OFDI images before and after RA are shown in the middle. The schema of the OFDI image is shown on the right. The red dot indicates wire, and the green circle indicates OFDI catheter. The yellow area indicates the actual ablated area (A-area). **A** The pre-RA image shows that in this target lesion, the OFDI catheter and wire are in contact (as shown in the white arrow in the schema). Post-RA OFDI image shows calcified plaque around the OFDI catheter on the pre-RA OFDI was debulked. It is speculated that the wire was initially located at the orange circle (upper schema), but the OFDI catheter itself altered the wire’s position to the red circle. Calcified plaque around the OFDI catheter and orange dot (presumably initial wire position) was debulked. **B** A pre-RA image of this target lesion reveals the OFDI catheter and wire are not in contact. The post-RA OFDI image shows a calcified plaque around the wire on the pre-RA OFDI that was debulked

Consequently, in cross sections where the OFDI catheter and wire were in contact, the median %Correct area was significantly higher in the OFDI catheter-based prediction method than in the wire-based prediction method (48.6% vs. 39.2%, *P* < 0.001, Fig. 3A). Furthermore, the median %Error angle was significantly lower in the OFDI catheter-based prediction method than in the wire-based prediction method (11.0% vs. 20.2%, *P* < 0.001, Fig. 3D). In contrast, in the cross sections where the OFDI catheter and wire were not in contact, the median %Correct area tended to be lower and the median %Error area was significantly higher in the OFDI catheter-based prediction method than in the wire-based prediction method (%Correct area: 41.0% vs. 52.5%, *P* = 0.091; Fig. 4A, %Error area: 68.1% vs. 42.2%, *P* < 0.001; Fig. 4B). Additionally, in these cross sections, the median %Correct angle was significantly lower and the median %Error angle was significantly higher in the OFDI catheter-based prediction method than in the wire-based prediction method (%Correct angle: 58.5% vs. 75.6%, *P* < 0.001, Fig. 4C); (%Error angle: 34.0% vs. 27.4%, *P* = 0.010, Fig. 4D). Figure 5 indicates representative cases with cross sections specifying whether or not the OFDI catheter and wire were in contact.

## Discussion

The main findings of this study can be summarized as follows: (1) OFDI image before RA can predict the amount and the location of debulked area of RA with median %Correct and %Error angles of 69.8% and 15.6%, respectively, and the median %Correct area and %Error areas of 47.8% and 41.6%, respectively; (2) short distance between wire and intima; OFDI catheter and wire, were independent predictors of good prediction. Whereas, long distance between the: OFDI catheter and wire; OFDI and intima; wire and intima, were independent predictors of irrelevant prediction of debulking effects of RA; (3) irrelevant ablation and over ablation were related to deep vessel injury and intimal flap outside the predicted area; and (4) in the cross sections where the OFDI catheter and wire were in contact, predictive accuracy was better with the OFDI catheter-based prediction method, while it was better with the wire-based prediction method in the cross sections where the OFDI catheter and wire were not in contact.

Severely calcified lesions remain an unsolved issue even in these days of improved outcomes of PCI. The unfavorable outcome of PCI in severely calcified lesions is mainly due to suboptimal peri-procedural results, including stent under-expansion, asymmetric stent expansion, and incomplete stent apposition [10, 11]. RA is widely used in recent PCIs for severely calcified lesions to solve these procedural problems. However, it has a potential risk of catastrophic complications such as perforation, coronary dissection, or slow-flow phenomenon [2]. Thus, several consensus documents have been published for the standardization of the procedure. For example, the contemporary European expert consensus document recommends initial usage of small burrs (1.25–1.5 mm) and preferably a burr/artery ratio of approximately 0.6 [2], while the North American Expert Review and Japanese clinical expert consensus document recommend burr/artery ratio of 0.4–0.6 [1, 12]. Nonetheless, these recommendations were based on angiographic images, which are sometimes equivocal.

OFDI may provide us with more accurate and detailed intravascular information than intravascular ultrasound [4, 13]. It enables us to evaluate the spatial distribution of calcified plaque and wire course in the vessel, which determines the indication of RA, appropriate burr size, and the lesion length to be debulked. For the first time in the previous retrospective study, we demonstrated that OFDI could predict the direction and the amount of RA using the same evaluation method as that of the present prospective study [6]. Specifically, the previous study demonstrated that the predictive accuracy for the amount of ablation was not high (the median %Correct and %Error areas were 43.1% and 64.2%, respectively, and the median %Correct and %Error volumes were 50.3% and 67.0%, respectively), whereas the predictive accuracy for the location of ablation area was feasible (%Correct angle was 55.3%, showing that in 87% of cross sections, at least part of the predicted region was ablated). However, the previous study had several limitations due to the retrospective design. First, the exclusion rate was extremely high (32 out of 58 lesions were excluded because OFDI could not pass before RA) and the permitted balloon size before RA was not strictly defined. In the current prospective protocol, pre-dilatation with a 2.0-mm balloon was permitted if the OFDI catheter could not pass the target lesion, therefore the exclusion rate was only 8.3% (5 out of 60 lesions), and the quality of pre-RA OFDI image was relatively standardized. Consequently, the predictive ability for the location of the ablation area improved (the median %Correct and %Error angles were 55.3%–69.8% and 38.3–15.6%, respectively), whereas the predictive ability for the amount of ablation area did not significantly improve (the median %Correct and %Error areas were 43.1–47.8% and 64.2–41.6%, respectively and the median %Correct and %Error volumes were 50.3–44.1% and 67.0–42.1%, respectively). We currently speculated that the Rota wire position gradually shifts toward or away from the vessel wall with a repetitive RA procedure; however, it is difficult to take its positional change into consideration for prediction. Therefore, the predictive accuracy for the amount of ablation area could not be so high. Second, several important procedural parameters, such as RA speed, burr size, debulking counts, or time, were not unified or missing in the previous study, which limited the investigation as a potential relationship between detailed procedural factors and predictive accuracy. In the current study, we unified the initial RA speed from 170,000 to 200,000 rpm and set the first burr size such that the burr to artery ratio < 0.7. Moreover, total debulking counts or total run times were recorded. As a result, none of the procedural factors were independently associated with the predictive accuracy of OFDI-based RA effect prediction. Third, only a few complications occurred in both the previous and the current studies. Therefore, the relationship between the predictive accuracy and the complication rate could not be directly evaluated. Nonetheless, the current study revealed that irrelevant ablation and over ablation was significantly associated with deep vessel injury extending beyond the media or intimal flap outside the P-area. This indicates that the predictive accuracy of OFDI might be related to peri-procedural complications, such as coronary perforation. Fourth, the previous study demonstrated that long distance between the: OFDI catheter and wire; OFDI and intima; wire and intima, were independently associated with irrelevant ablation [6], which was consistent with the current study. In this prospective study, every effort was undertaken to minimize deflection in all cases, nonetheless, the results were consistent with that of the retrospective one. This indicates that the distance between OFDI catheter and wire is an important factor associated with predictive accuracy, which could be an unavoidable, inherent problem in some cases. In the current study, we discovered that in the cross-section where the OFDI catheter and wire were not in contact, the predictive accuracy was superior in wire-based analysis. The reasons for the results are discussed using representative cases (Fig. 5). In Fig. 5A, where the OFDI catheter and wire were in contact, calcified plaque around the OFDI catheter, and not the wire, was debulked. Since the OFDI catheter is a relatively stiff, short monorail system [14], and has a size of 0.9-mm, the original position of the wire was possibly altered by insertion of the OFDI catheter. In other words, the location of the wire on OFDI image is always affected by the OFDI catheter itself. Therefore, the actual location of the wire before OFDI catheter insertion cannot be evaluated with OFDI images. Therefore, even though the RA system is an over-the-wire device, OFDI catheter-based prediction method is more accurate than wire-based prediction method in most cross sections. In contrast, as in Fig. 5B where the OFDI catheter and wire were separated, calcified plaque around the wire was debulked. Since the OFDI catheter and wire follow the same passage, there may be a certain bias in the cases where the OFDI catheter and wire are separated. In such cases, it would be more accurate to predict that debulking effects will occur around the wire.

### Limitations

This study has several limitations. First, although this is a prospective study, several procedural options such as wire selection and guide-catheter type were based on the discretion of the operator. Although floppy wire use was strongly associated with good prediction in the previous study, this differed in the present study. It is possibly due to a bias that operators willingly chose floppy wire in the cases which were considered irrelevantly ablated. Second, because of the relatively small sample size in this study, our findings should carefully be applied to clinical practice. Third, we evaluated cross-sectional findings of deep vessel injury or intimal flap outside the P-area as a surrogate for peri-procedural complication owing to the low complication rate, however, it remains uncertain whether the predictive accuracy is related to the incidence of complication. Fourth, there is a difference in that RA system runs anterogradely whereas OFDI catheter scans retrogradely, which can have some negative impact on the predictive accuracy. Although not investigated in the current study, anterograde IVUS scanning may be useful to improve the predictive accuracy. Last, there might be a bias because this is a cross-sectional analysis and the number of cross sections with the debulked region varies among individuals.

## Conclusions

The prospective study demonstrated that OFDI image before RA can predict the amount and the location of the debulked area of RA to a certain extent. The predictive accuracy may be improved by focusing on the position of both the OFDI catheter and wire.

## Supplementary Information

Below is the link to the electronic supplementary material.

Supplementary file1 (DOCX 1554 KB)

Supplementary file2 (PPTX 36 KB)

Supplementary file3 (PPTX 5980 KB)

Supplementary file4 (PPTX 70 KB)

Supplementary file5 (PPTX 94 KB)

## Data Availability

The deidentified participant data will not be shared.
