# Supplementary material for: Prediction of the debulking effect of rotational atherectomy using optical frequency domain imaging: a prospective study
Source: Cardiovasc Interv Ther. 2023 Apr 5;38(3):316–26. doi: 10.1007/s12928-023-00928-9 (PMC10247835; doi:10.1007/s12928-023-00928-9)

**SUPPLEMENTARY DOCUMENT**

**Prediction of the debulking effect of rotational atherectomy**

**using optical frequency domain imaging: a prospective study**

**Cardiovascular Intervention and Therapeutics**

Tomoyo Hamana, MD^1^, Hiroyuki Kawamori, MD, PhD^1^, Takayoshi Toba, MD, PhD^1^, Makoto Nishimori, MD, PhD^1,2^, Kosuke Tanimura, MD, PhD^1^, Shunsuke Kakizaki, MD^1^, Koichi Nakamura, MD^1^, Daichi Fujimoto MD, PhD^1^, Satoru Sasaki, MD^1^, Yuto Osumi, MD^1^, Masayoshi Fujii, MD^1^, Seigo Iwane, MD^1^, Tetsuya Yamamoto, MD^1^, Shota Naniwa, MD^1^, Yuki Sakamoto, MD^1^, Yuta Fukuishi, MD^1^, Koshi Matsuhama, MD^1^, Ken-ichi Hirata, MD, PhD^1^, Hiromasa Otake, MD, PhD^1^*

^1^ Division of Cardiovascular Medicine, Department of Internal Medicine, Kobe University Graduate School of Medicine, Kobe, Japan

^2^ Division of Epidemiology, Kobe University Graduate School of Medicine, Kobe, Japan

*Corresponding Author:

Hiromasa Otake, MD, PhD

E-mail address: hotake@med.kobe-u.ac.jp

**TABLE OF CONTENTS**

**Supplementary Table 1**:

Univariable and multivariable logistic regression analyses according to the lesion-level good prediction group p 3

**Supplementary Table 2**:

Univariable and multivariable logistic regression analyses according to the lesion-level irrelevant ablation group p 4

**Supplementary Figure legends**

**Supplementary Figure 1**: Patient flowchart. p 5

**Supplementary Figure 2:** Representative cases of four groups p 6

**Supplementary Figure 3**: Distributions of post-RA findings among four groups. p 7

**Supplementary Figure 4**: ROC curve of the minimum distance between the OFDI catheter and wire predicting for cross-section with irrelevant ablation group. p 8

**Supplementary Table 1**: **Univariable and multivariable logistic regression analyses according to the lesion-level good prediction group**

| Variables | Univariable | | | Multivariable | | |
| --- | --- | --- | --- | --- | --- | --- |
|  | OR | 95% CI | *P* value | OR | 95% CI | *P* value |
| LAD | 4.67 | 1.15 – 18.9 | 0.031 | 4.62 | 1.11 – 19.3 | 0.036 |
| Proximal site | 1.94 | 0.57 – 6.61 | 0.29 |  |  |  |
| Floppy wire | 2.72 | 0.66 – 11.2 | 0.16 |  |  |  |
| Judkins type guiding catheter | 1.36 | 0.34 – 5.46 | 0.66 |  |  |  |
| Total run time | 1.00 | 0.99 – 1.01 | 0.60 |  |  |  |
| Total debulking counts | 1.00 | 0.95 – 1.05 | 1.00 |  |  |  |
| Pre minimum lumen area | 0.53 | 0.24 – 1.16 | 0.11 | – | – | – |
| OR, odds ratio; CI, confidence interval; LAD, left anterior descending. | | | | | | |

**Supplemental Table 2. Univariable and multivariable logistic regression analyses according to the lesion-level irrelevant ablation group**

| Variables | Univariable | | | Multivariable | | |
| --- | --- | --- | --- | --- | --- | --- |
|  | OR | 95% CI | *P* value | OR | 95% CI | *P* value |
| RCA | 4.64 | 1.10 – 19.5 | 0.036 | 5.25 | 1.17 – 23.7 | 0.031 |
| Proximal site | 0.93 | 0.29 – 2.99 | 0.91 |  |  |  |
| Floppy wire | 0.35 | 0.10 – 1.17 | 0.089 | – | – | – |
| Judkins type guiding catheter | 1.36 | 0.34 – 5.46 | 0.66 |  |  |  |
| Total run time | 1.00 | 0.99 – 1.01 | 0.63 |  |  |  |
| Total debulking counts | 0.99 | 0.94 – 1.04 | 0.63 |  |  |  |
| Pre minimum lumen area | 1.36 | 0.79 – 2.32 | 0.26 |  |  |  |
| OR, odds ratio; CI, confidence interval; RCA, right coronary artery. | | | | | | |

**Supplemental Figure 1. Patient flowchart**

OFDI, optical frequency domain imaging; PCI, percutaneous coronary intervention; RA, rotational atherectomy


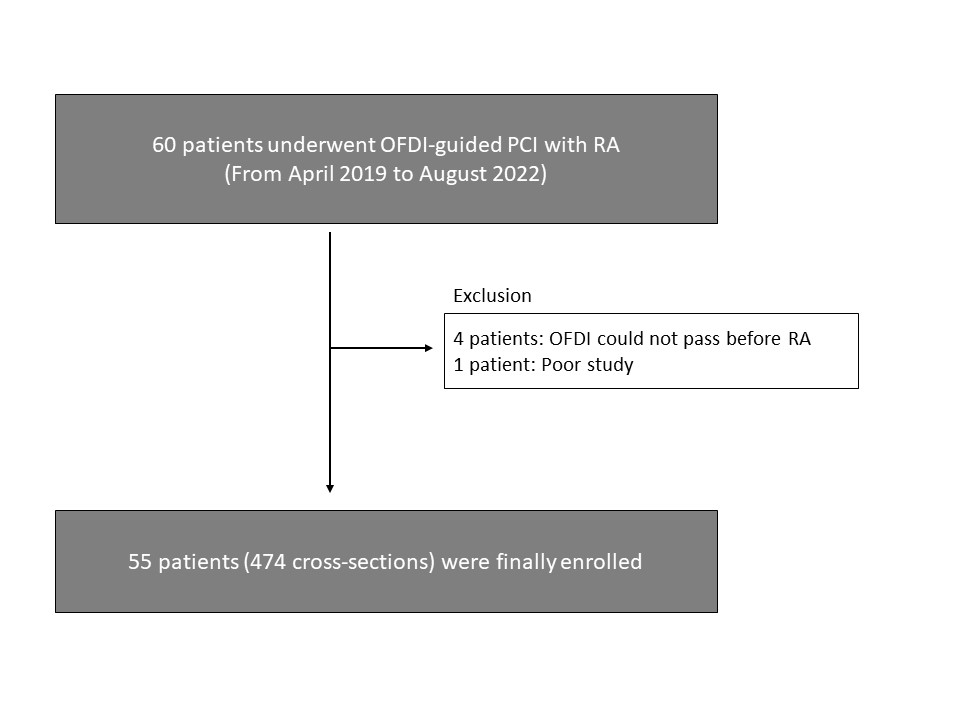


**Supplementary Figure 2: Representative cases of four groups**

(A) Good prediction group. (B) Over ablation group. (C) Insufficient group. (D) Irrelevant ablation group.

Angiography images with (upper left) and without contrast (upper right) are shown. White triangles indicate target lesion. OFDI images before (lower left) and after RA (lower right) are shown.

OFDI, optical frequency domain imaging; RA, rotational atherectomy


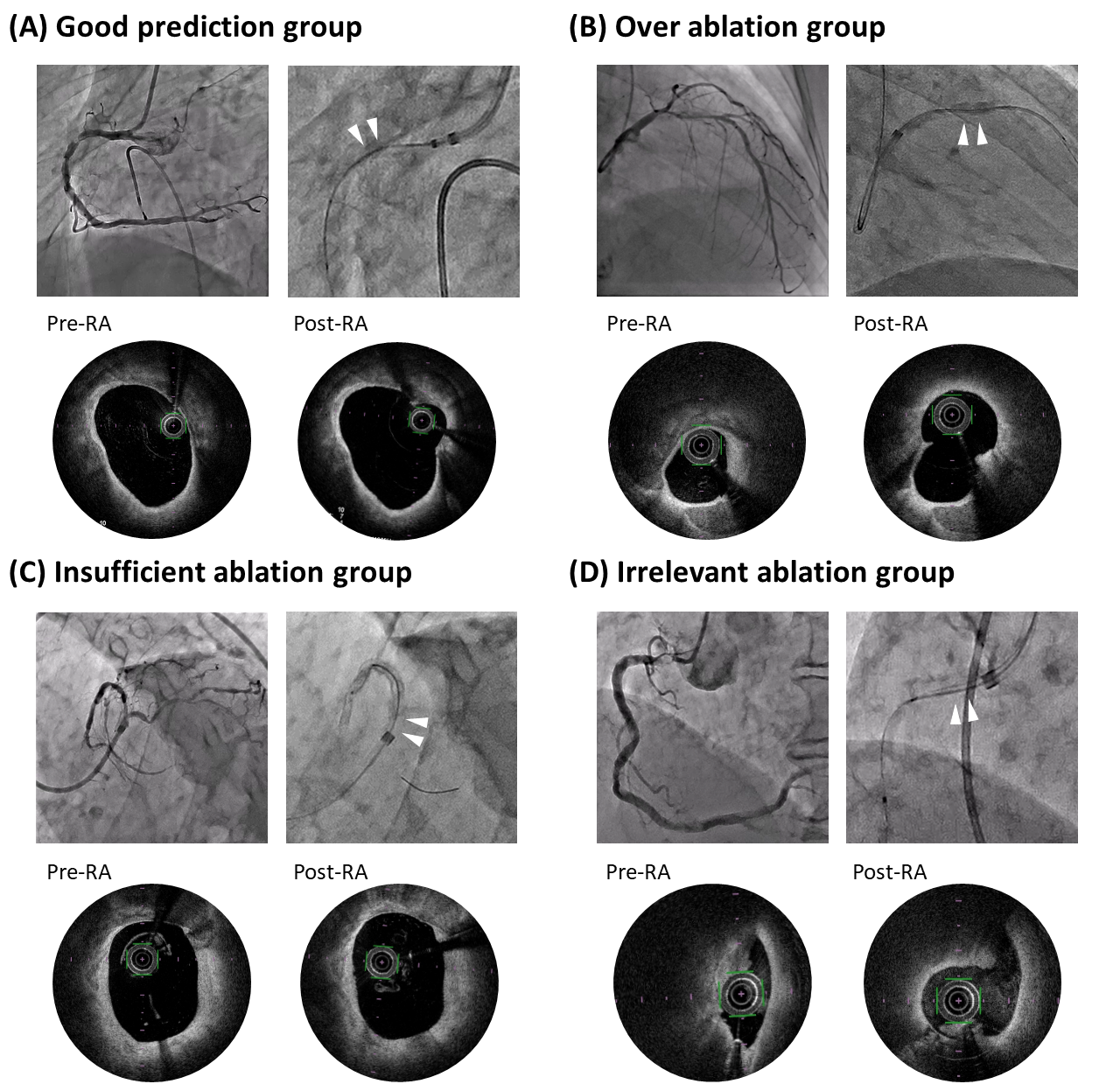


**Supplemental Figure 3. Distributions of post-RA findings among four groups.**

(A) Presence of deep vessel injury extending beyond the media. (B) Presence of intimal flap outside the P-area. The cross-section with deep vessel injury extending beyond the media is more frequently observed in the over ablation group and the irrelevant ablation group than in the other groups (χ^2^ test: *P*=0.008). The cross-section with an intimal flap outside the P-area is more frequently observed in the over ablation group and the irrelevant ablation group than in the other groups (χ^2^ test: *P*<0.001).

One asterisk (*) indicates *P*<0.05, and three asterisks (***) indicate *P*<0.01.


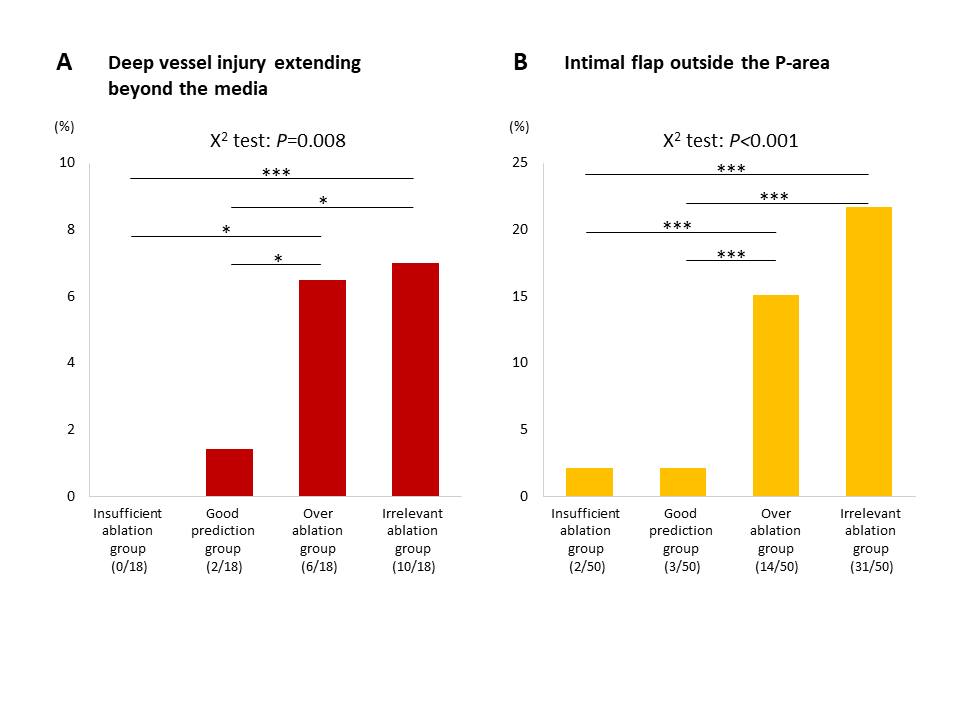


**Supplemental Figure 4. ROC curve of the minimum distance between the OFDI catheter and wire predicting for cross-section with irrelevant ablation group.**

ROC, receiver operating characteristic; OFDI, optical frequency domain imaging; AUC, area under the curve; CI, confidence interval


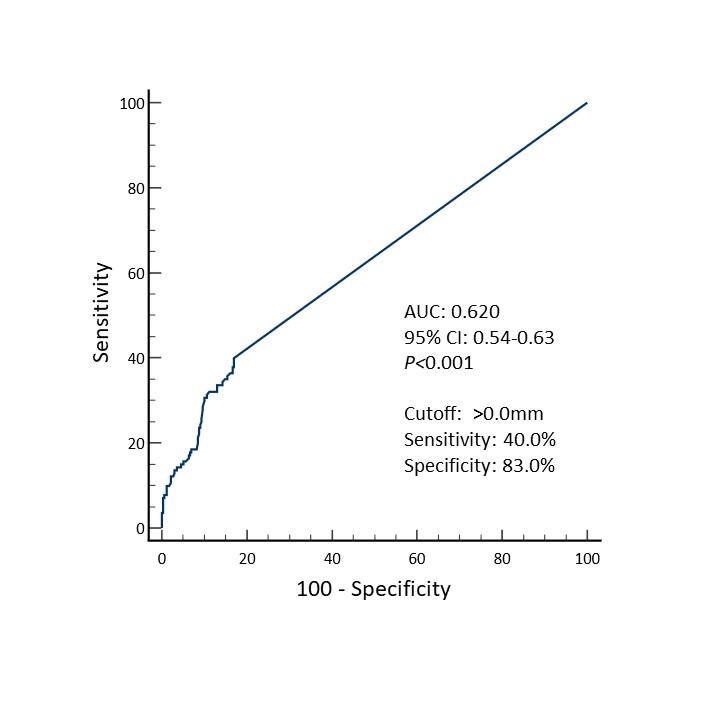

Supplement: Supplementary file 1 — Supplementary file1 (DOCX 1554 KB) [file 12928_2023_928_MOESM1_ESM.docx]
