# Supplementary material for: Prediction of the debulking effect of rotational atherectomy using optical frequency domain imaging: a prospective study
Source: Cardiovasc Interv Ther. 2023 Apr 5;38(3):316–26. doi: 10.1007/s12928-023-00928-9 (PMC10247835; doi:10.1007/s12928-023-00928-9)
Supplement: Supplementary file 2 — Supplementary file2 (PPTX 36 KB) [file 12928_2023_928_MOESM2_ESM.pptx]

## Slide 1
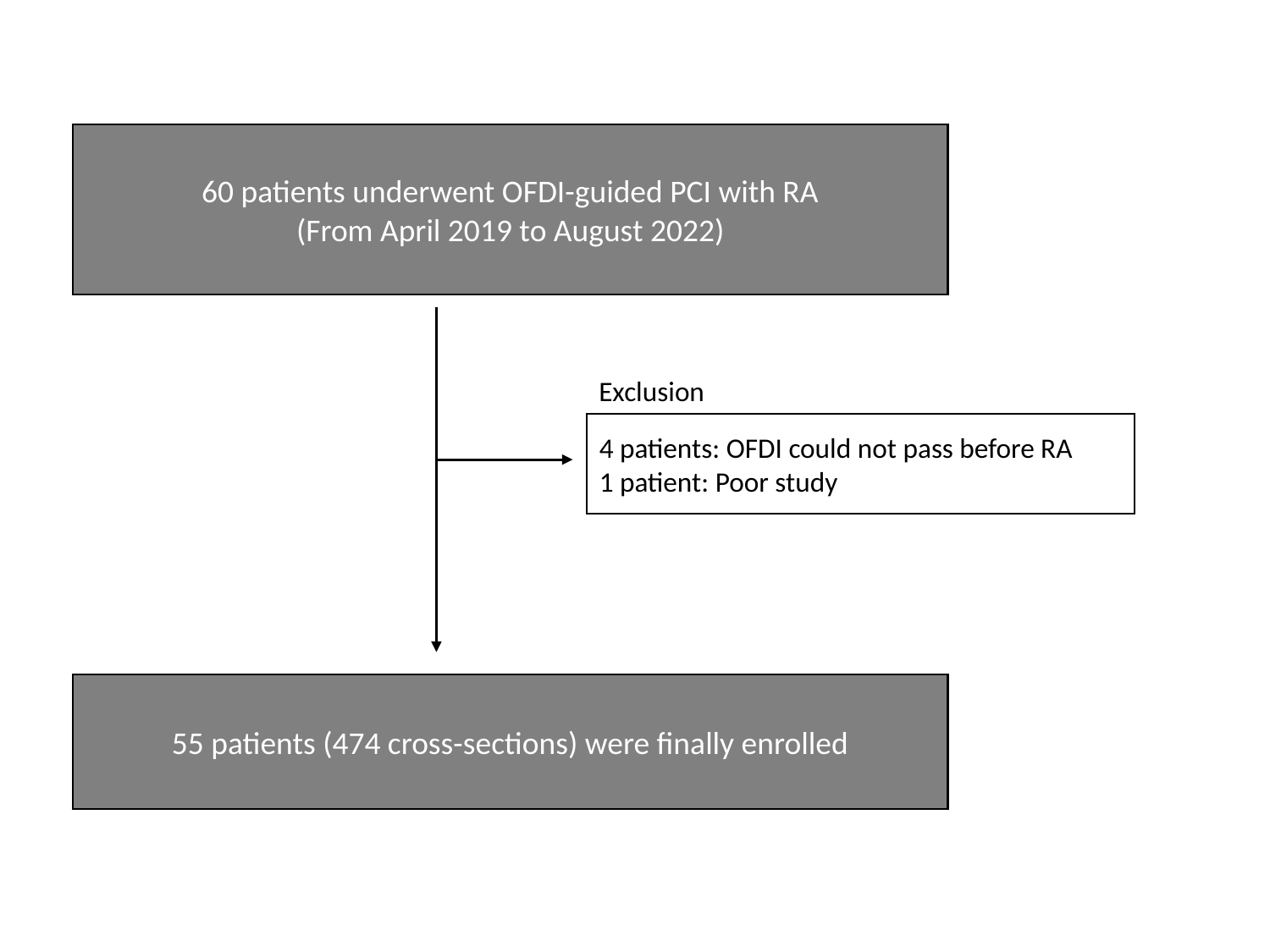

60 patients underwent OFDI-guided PCI with RA
(From April 2019 to August 2022)
Exclusion
4 patients: OFDI could not pass before RA
1 patient: Poor study
55 patients (474 cross-sections) were finally enrolled
