# Supplementary material for: Prediction of the debulking effect of rotational atherectomy using optical frequency domain imaging: a prospective study
Source: Cardiovasc Interv Ther. 2023 Apr 5;38(3):316–26. doi: 10.1007/s12928-023-00928-9 (PMC10247835; doi:10.1007/s12928-023-00928-9)
Supplement: Supplementary file 3 — Supplementary file3 (PPTX 5980 KB) [file 12928_2023_928_MOESM3_ESM.pptx]

## Slide 1
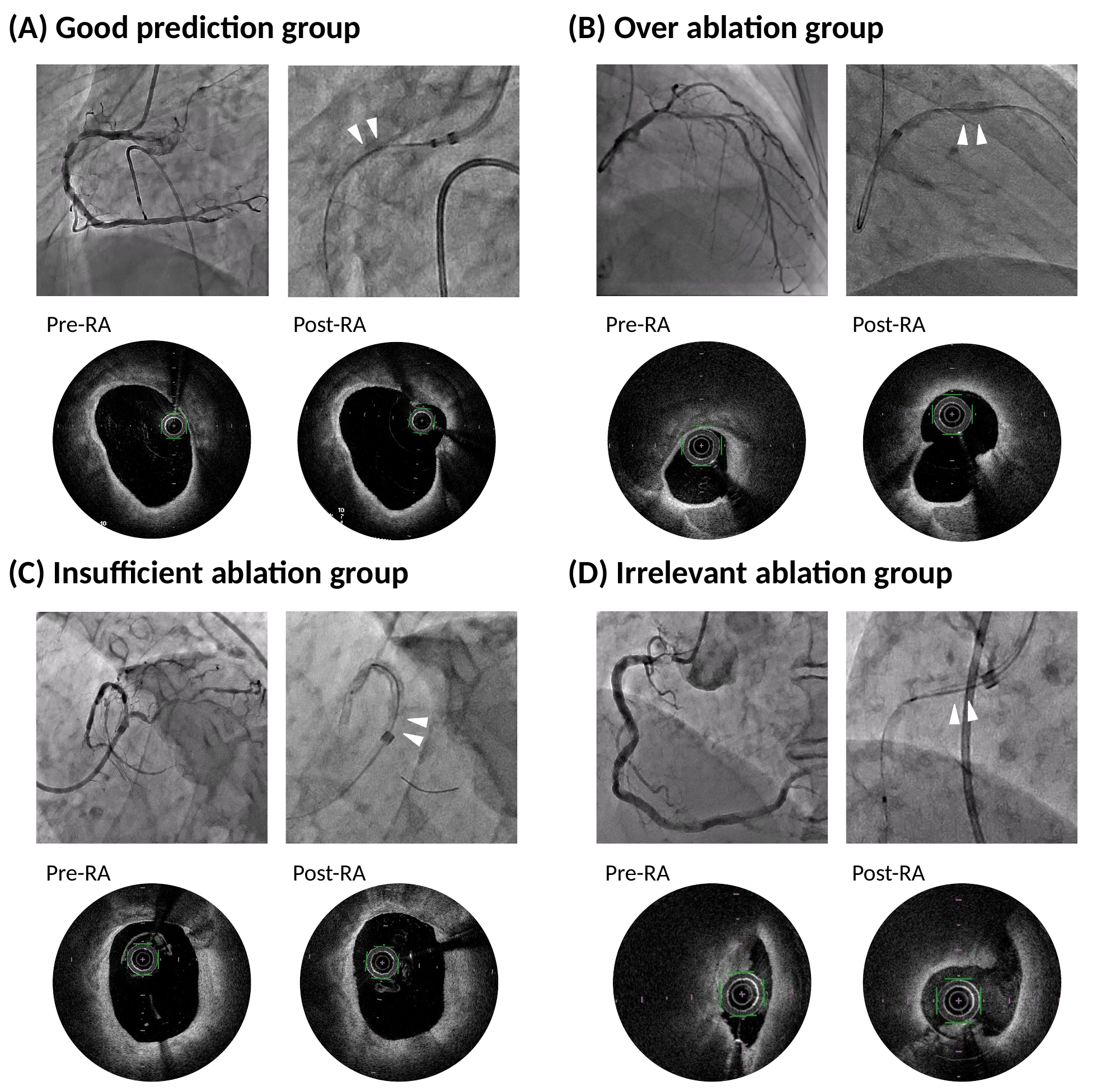

(B) Over ablation group
(A) Good prediction group
Pre-RA
Post-RA
Pre-RA
Post-RA
(C) Insufficient ablation group
(D) Irrelevant ablation group
Pre-RA
Post-RA
Pre-RA
Post-RA
