# Supplementary material for: Prediction of the debulking effect of rotational atherectomy using optical frequency domain imaging: a prospective study
Source: Cardiovasc Interv Ther. 2023 Apr 5;38(3):316–26. doi: 10.1007/s12928-023-00928-9 (PMC10247835; doi:10.1007/s12928-023-00928-9)
Supplement: Supplementary file 4 — Supplementary file4 (PPTX 70 KB) [file 12928_2023_928_MOESM4_ESM.pptx]

## Slide 1
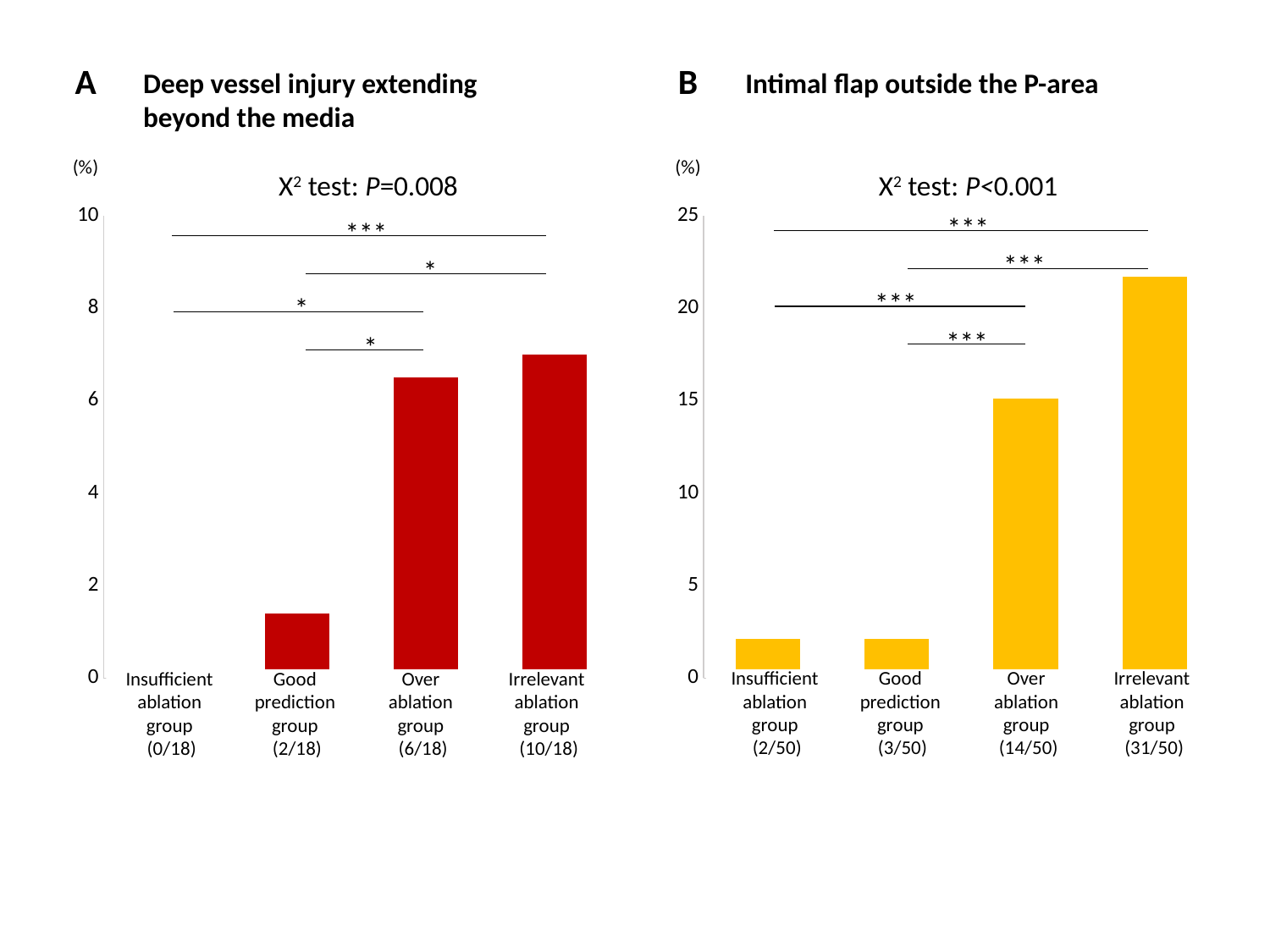

A
B
(%)
(%)
Χ2 test: P=0.008
Χ2 test: P<0.001
### Chart
| Category | 系列 1 |
|---|---|
| Insufficient ablation group | 0.0 |
| Good prediction group | 1.4 |
| Over ablation group | 6.5 |
| Irrelevant ablation group | 7.0 |
### Chart
| Category | 系列 1 |
|---|---|
| Insufficient ablation group | 2.1 |
| Good prediction group | 2.1 |
| Over ablation group | 15.1 |
| Irrelevant ablation group | 21.7 |Irrelevant ablation group
 (31/50)
Insufficient ablation group
 (2/50)
Good prediction group
 (3/50)
Over ablation group
 (14/50)
Irrelevant ablation group
 (10/18)
Insufficient ablation group
 (0/18)
Good prediction group
 (2/18)
Over ablation group
 (6/18)
Deep vessel injury extending beyond the media
Intimal flap outside the P-area
***
***
***
*
***
*
***
*
