# Supplementary figures and images for: Prediction of the debulking effect of rotational atherectomy using optical frequency domain imaging: a prospective study
Source: Cardiovasc Interv Ther. 2023 Apr 5;38(3):316–26. doi: 10.1007/s12928-023-00928-9 (PMC10247835; doi:10.1007/s12928-023-00928-9)

## Slide 1
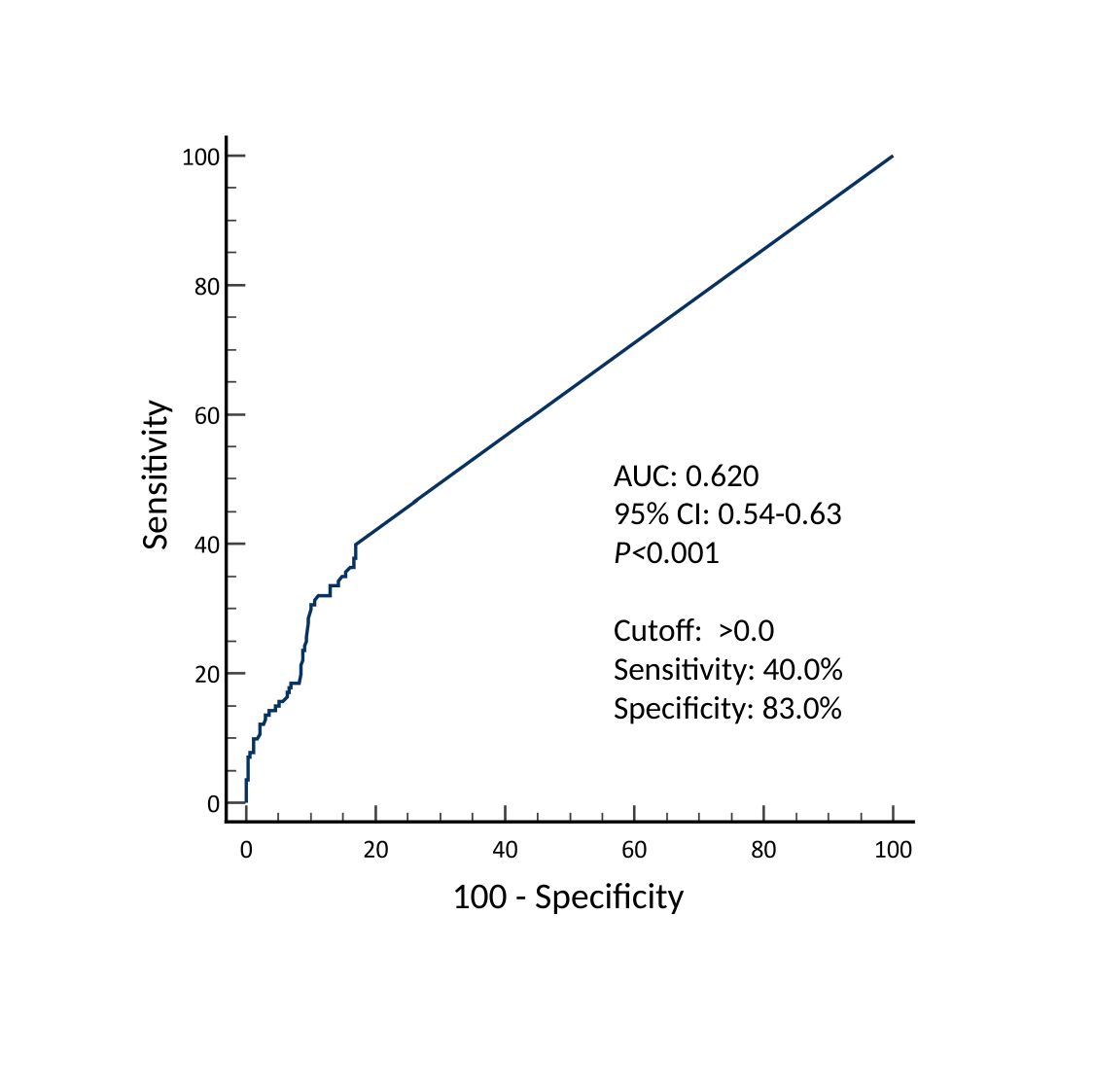

Sensitivity
100 - Specificity
AUC: 0.620
95% CI: 0.54-0.63
P<0.001
Cutoff: >0.0
Sensitivity: 40.0%
Specificity: 83.0%

Supplement: Supplementary file 5 — Supplementary file5 (PPTX 94 KB) [file 12928_2023_928_MOESM5_ESM.pptx]
